# Role of age and birth month in infants hospitalized with RSV‐confirmed disease in the Valencia Region, Spain

**DOI:** 10.1111/irv.12937

**Published:** 2021-11-24

**Authors:** Ainara Mira‐Iglesias, Clarisse Demont, F. Xavier López‐Labrador, Beatriz Mengual‐Chuliá, Javier García‐Rubio, Mario Carballido‐Fernández, Miguel Tortajada‐Girbés, Juan Mollar‐Maseres, Germán Schwarz‐Chavarri, Joan Puig‐Barberà, Javier Díez‐Domingo, for the Valencia Hospital Network for the Study of Influenza and other Respiratory Viruses

**Affiliations:** ^1^ Área de Investigación en Vacunas Fundación para el Fomento de la Investigación Sanitaria y Biomédica de la Comunitat Valenciana (FISABIO‐Public Health) Valencia Spain; ^2^ RSV Medical Evidence Generation Sanofi Pasteur Lyon France; ^3^ Laboratorio de Virología Área de Genómica y Salud. Fundación para el Fomento de la Investigación Sanitaria y Biomédica de la Comunitat Valenciana (FISABIO‐Public Health) Valencia Spain; ^4^ Consorcio de Investigación Biomédica de Epidemiología y Salud Pública (CIBER‐ESP) Instituto de Salud Carlos III Madrid Spain; ^5^ Preventive Medicine Hospital General Universitario de Castellón Castellón de la Plana Spain; ^6^ Medicine Department Universidad CEU Cardenal Herrera Castellón de la Plana Spain; ^7^ Pediatric Pneumology and Allergy Unit Hospital Universitario Doctor Peset Valencia Spain; ^8^ Preventive Medicine Hospital Universitario y Politécnico La Fe Valencia Spain; ^9^ Out‐of‐Hospital Family and Community Medicine Hospital General Universitario de Alicante Alicante Spain

**Keywords:** hospitalizations, infants, respiratory syncytial virus, surveillance

## Abstract

**Background:**

RSV is the leading cause of hospital admissions in infants and the principal cause of bronchiolitis in young children. There is a lack of granular data on RSV‐associated hospitalization per season using laboratory confirmed results. Our current study addresses this issue and intends to fill this gap.

**Methods:**

The study was conducted from 2014 through 2018, in 4 to 10 hospitals in the Valencia Region, Spain. Infants included in this study were admitted in hospital through the Emergency Department with a respiratory complaint and tested by RT‐PCR for RSV in a central laboratory.

**Results:**

Incidence rates of RSV‐associated hospitalization varied by season and hospital. Overall, the highest incidence rates were observed in 2017/2018. RSV‐associated hospitalization was highest in infants below 3 months of age and in those born before or at the beginning of the RSV season. Almost 54% of total infants hospitalized with laboratory confirmed RSV were found to be born outside the season, from April to October. The RSV positivity rate by ICD‐10 discharged codes varied by season and age with results from 48% to 57% among LRI (J09‐J22).

**Conclusion:**

The study was instrumental in bringing forth the time unpredictability of RSV epidemics, the critical impact of age, and the comparable distribution of RSV‐associated hospitalization in infants born on either side of the RSV season. These data could help in better characterization of the population that drives the healthcare burden and is crucial for the development of future immunization strategies, especially with upcoming vaccines in against RSV.

## 1 INTRODUCTION

Each year, nearly 33 million cases of acute lower respiratory tract infection (ALRTI) associated with Respiratory Syncytial virus (RSV) are diagnosed in children under 5 years old.^1^, ^2^ RSV is the principal cause of bronchiolitis in young children^3^ and is globally the leading cause of hospital admissions in infants.^4^, ^5^ Although mortality due to RSV is very low among children in high‐income countries,^6^ yet it plays an important role in the hospital resources utilization.^7^, ^8^ RSV is responsible for nearly 16 times more infant hospitalizations than influenza.^9^

In temperate climates, RSV circulation starts generally around November and end in March–April.^2^, ^10^

Age, prematurity, birth close to the start of the RSV season, and presence of chronic conditions have been commonly identified as potential risk factors for RSV‐associated disease.^7^, ^11^, ^12^ However, by the age of two, almost all children are infected by RSV.^13^

Despite the high unmet medical need, a solution to protect all infants at risk to develop an RSV‐associated disease is not yet available, but several candidates are expected to be licensed in coming years. Therefore, understanding the disease burden by month of age and by month of birth to determine who will benefit the most from these vaccination or monoclonal antibodies strategies is valuable to support future immunization policies and recommendations. Usually, burden‐of‐disease data are estimated using International Statistical Classification of Diseases and Related Health Problems (ICD) diagnosis codes on syndromic surveillance^14^ or hospital discharges data.^15^ Despite strengths of these studies, they usually do not report laboratory confirmed RSV information. To overcome this issue, we conducted a prospective active‐surveillance study during 4 years in Valencia Region, Spain. The objective of this study was to better quantify the incidence of RSV‐associated hospitalized disease by season, age, and month of birth. In addition, we aimed to describe the clinical presentation of the RSV‐associated disease and to determine the risk factors of infants hospitalized with laboratory confirmed RSV.

## 2 METHODS

### 2.1 Study population

The study was conducted from 2014 through 2018 in 4 to 10 hospitals (depending on the season) in the Valencia Region: at Hospital General Universitario de Castellón (Castellón, Spain), Hospital Universitario de La Plana (Villarreal, Spain), Hospital Universitario y Politécnico La Fe (Valencia, Spain), Hospital Universitario Doctor Peset (Valencia, Spain), Hospital Universitario de La Ribera (Alzira, Spain), Hospital Universitario San Juan de Alicante (San Juan de Alicante, Spain), Hospital General Universitario de Elda (Elda, Spain), Hospital General Universitario de Alicante (Alicante, Spain), and Hospital Universitario del Vinalopó (Elche, Spain). The catchment area of these hospitals was well defined; they covered 21% (4 hospitals) to 46% (10 hospitals) of total inhabitants of the Valencia Region. From November to March/April every year, except in 2017/2018 season (from September to June), the active surveillance of RSV was set up. RSV circulation period was defined as the weeks between the first of at least two consecutive weeks with two or more RSV cases and the week prior to the first of at least two consecutive weeks without RSV cases. Hospitalized patients from all age groups meeting inclusion criteria were enrolled in the study. For the current publication, we considered only infant population aged <1 year.

### 2.2 Study design

The methodology of the active‐surveillance network has been already described in previous publications.^16^, ^17^, ^18^ Full‐time dedicated nurses screened consecutive hospitalized patients discharged from the Emergency Department with a diagnosis possibly related to a respiratory infection (Table S1). Patients were included in the study if they were resident in the catchment area of one of the participating hospitals, non‐institutionalized, and not discharged from a previous admission in the last 30 days. The onset of symptoms that led to hospitalization was required to be 7 days prior to admission, and patients had to be in hospital between 8 and 48 h before their inclusion in the study. Infants under 1 month of age who left the hospital after delivery with no incidents (who were not admitted after birth in the neonatal unit) and who were subsequently hospitalized after a period of 1 week in the community were susceptible to be included in the study.

The Ethics Research Committee of the Dirección General de Salud Pública‐Centro Superior de Investigación en Salud Pública (DGSP‐CSISP) approved the protocol of the study. All caregivers signed written informed consent prior to inclusion of their infants in the study.

### 2.3 Laboratory procedures

Nasopharyngeal and nasal (FLOQSwabs, Copan, Italy) swabs were obtained within the first 48 h of admission from each patient fulfilling the inclusion criteria. Both swabs were combined in a tube of viral transport media (Copan, Italy) and frozen between −50°C and −20°C until shipped refrigerated to a centralized Virology laboratory at FISABIO‐Public Health. One third of the viral transport media volume was used to extract total nucleic acids using an automated silica‐based method (Nuclisens Easy‐Mag, BioMérieux, Lyon, France). Extracted nucleic acids were tested for RSV, influenza, and other respiratory viruses (a total of 19) by multiplex real‐time reverse transcription‐polymerase chain reaction (RT‐PCR), following WHO protocols^19^ with the qScript XLT One‐Step RT‐qPCR ToughMix (Quanta BioSciences, MD, USA) in a Lightcycler 480II apparatus (Roche Diagnostics, Spain).

### 2.4 Statistical analysis

#### 2.4.1 RSV‐associated hospitalization incidence rates by season

We calculated the RSV hospitalization incidence rates per 100,000 infants‐season overall and by hospital. The catchment area of each participating hospital along the different seasons was considered as the denominator. The RSV circulation period was estimated by epidemiological weeks, using EPIWEEK STATA module.

#### 2.4.2 RSV‐associated hospitalization incidence rates by age and birth month

According to age (0 to 11 months) and birth months (January to December), we calculated the RSV hospitalization incidence rates per 100,000 infants‐season. The numerator was the number of RSV cases by age or by birth month for each season. The denominator was estimated by dividing the catchment population under 1 year old by 12. Due to the different duration of the seasons and to allow comparisons between them, the RSV hospitalization incidence rates were provided per 100,000 infants‐week and per 100,000 infants‐month (restricted to the RSV circulation period).

#### 2.4.3 Risk factors of infants hospitalized with laboratory confirmed RSV

Comparison between RSV positive and RSV negative hospitalizations was conducted based on the following parameters: (1) birth month, (2) age (in months), (3) prematurity (<29 weeks, 29 to <37 weeks, ≥37 weeks), (4) associated comorbidities (chronic cardiovascular disease, chronic obstructive pulmonary disease [COPD], bronchitis, or any other chronic respiratory disease except asthma, anemia, and renal impairment), (5) contact with kids, and (6) kindergarten/school attendance and exposure to tobacco. Either Pearson Chi‐squared or Fisher's exact tests were performed, as appropriate, to obtain *p* values.

#### 2.4.4 Impact of age on RSV‐associated hospitalization

A negative binomial regression, a generalization of the Poisson regression model that addresses the over‐dispersion issue, was performed to assess the impact of age (≤3 months, >3 months) on RSV hospitalization incidence rates, after adjusting by calendar month at hospital admission (restricted from November to March), hospital and season. The population denominator was included as an offset. The adjusted relative risks (aRR) and their 95% confidence intervals (CIs) were provided.

#### 2.4.5 RSV‐associated disease—RSV positivity rate according to ICD‐10 discharge diagnoses by season and age

Hospital discharge information using the 10th revision of ICD diagnosis codes (ICD‐10) was retrieved for each infant included in the study. RSV‐associated disease was defined based on the following ICD‐10 codes recorded at hospital discharge: Lower Respiratory Infection (LRI): J09‐J22, bronchiolitis: J21 and pneumonia: J12‐J18. These outcomes were described by season and age group (<3 months, 3 to 5 months, and 6 to 11 months). Laboratory confirmed RSV results were used to determine RSV positivity rates for each disease outcome.

All statistical analyses were carried out in Stata version 14 (StataCorp, College Station, Texas) and R (Viena, Austria). All probabilities were two‐tailed, and *p* values <0.05 were considered significant.

## 3 RESULTS

### 3.1 Description of the study population from 2014/2015 to 2017/2018

**FIGURE 1:**
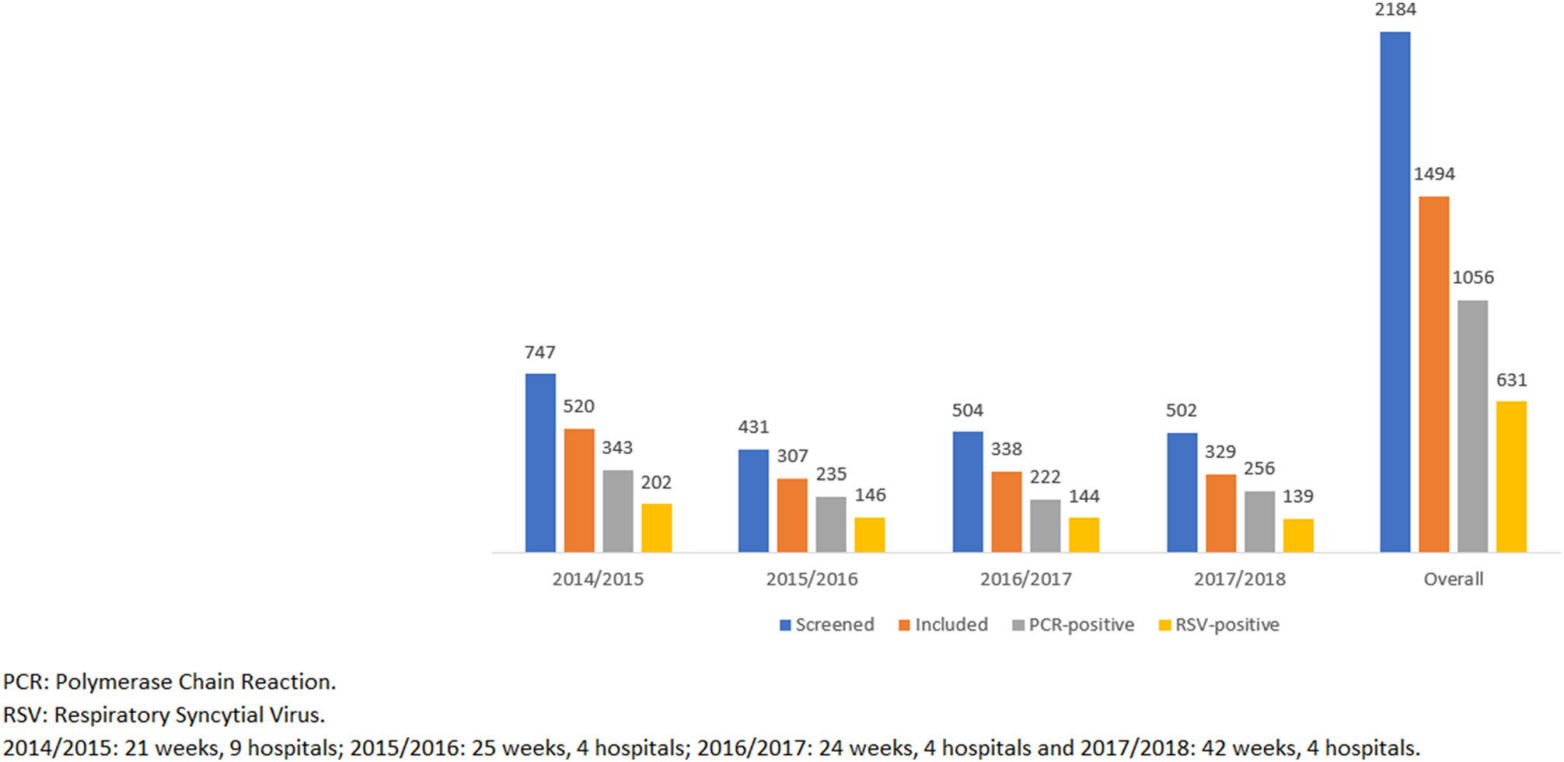
Screened patients, included patients, PCR‐positive patients, and RSV‐positive patients by season and overall. Infants hospitalized in the VAHNSI network, Valencia Region, Spain

The total infant catchment population varied from 8,726 to 18,414 depending on the number of hospitals included in the study. Throughout the four seasons, 2,184 infants with respiratory symptoms were identified at hospitals. A total of 1,494 (68.41%) were included in the study. Among those, 631 (42.24%) were RSV positive (Table 1; Figure 1). Re‐infection was not detected in infants included in the study. Most of RSV‐associated hospitalizations (81%) occurred in otherwise healthy infants. Out of the 631 RSV positive patients, we subtyped 574 samples of which 64% were found to be RSV A (by season: 67%, 78%, 47%, and 65%, data not shown). Eighty three (13% of total RSV positive infants) out of the 631 RSV positive patients had co‐infections, mainly rhinovirus (46%), coronavirus (25%), and bocavirus (14%) (data not shown).

**TABLE 1:** Study population, screened patients, included patients, PCR‐positive patients, and RSV‐positive patients by season and hospital

Surveillance period	Hospital	Population	Screened	Included	PCR‐positive	RSV‐positive						
						RSV surveillance period		RSV circulation period				
		*N*	*N*	*N*	*N*	*N*	Rate x100,000 infants‐season^a^	N	Weeks	Persons‐time (in weeks)	Rate ×100,000 infants‐week	Rate ×100,000 infants‐month^b^
**2014/2015** (21 weeks, 4.8 months)	General Castellón	2,469	174	124	91	54	2187.12	53	16	39,504	134.16	580.93
	La Plana	1,707	83	67	51	39	2284.71	39		27,312	142.79	618.30
	La Fe	1,672	64	53	28	19	1136.36	19		26,752	71.02	307.53
	Dr Peset	3,017	77	57	31	13	430.89	13		48,272	26.93	116.61
	La Ribera	2,280	121	83	59	39	1710.53	39		36,480	106.91	462.91
	San Juan	1,629	27	21	12	5	306.94	5		26,064	19.18	83.06
	Elda	1,690	59	50	30	15	887.57	15		27,040	55.47	240.20
	General Alicante	2,391	115	44	29	13	543.71	11		38,256	28.75	124.50
	Vinalopó	1,559	27	21	12	5	320.72	5		24,944	20.04	86.79
	**Overall**	**18,414**	**747**	**520**	**343**	**202**	**1,096.99**	**199**		**294,624**	**67.54**	**292.46**
2015/2016 (24 weeks, 5.5 months)	General Castellón	2,384	166	130	101	59	2474.83	59	20	47,680	123.74	535.80
	La Fe	2,597	75	48	32	24	924.14	24		51,940	46.21	200.08
	Dr Peset	2,147	77	68	55	33	1537.03	32		42,940	74.52	322.68
	General Alicante	2,428	113	61	47	30	1235.58	30		48,560	61.78	267.50
	**Overall**	**9,556**	**431**	**307**	**235**	**146**	**1,527.84**	**145**		**191,120**	**75.87**	**328.51**
**2016/2017** (23 weeks, 5.3 months)	General Castellón	2,288	136	109	65	36	1,573.43	36	17	38,896	92.55	400.76
	La Fe	2,434	73	52	36	25	1,027.12	24		41,378	58.00	251.15
	Dr Peset	2,098	118	84	55	33	1,572.93	33		35,666	92.53	400.63
	General Alicante	2,327	177	93	66	50	2,148.69	50		39,559	126.39	547.28
	**Overall**	**9,147**	**504**	**338**	**222**	**144**	**1,574.29**	**143**		**155,499**	**91.96**	**398.20**
**2017/2018** (41 weeks, 9.5 months)	General Castellón	2,279	163	127	100	52	2,281.70	50	19	43,301	115.47	499.99
	La Fe	2,428	76	56	42	18	741.35	17		46,132	36.85	159.56
	Dr Peset	1,685	86	39	29	19	1,127.60	19		32,015	59.35	256.97
	General Alicante	2,334	177	107	85	50	2,142.25	49		44,346	110.49	478.44
	**Overall**	**8,726**	**502**	**329**	**256**	**139**	**1,592.94**	**135**		**165,794**	**81.43**	**352.58**

*Note*: Infants hospitalized in the VAHNSI network, Valencia Region, Spain.

Abbreviations: PCR, Polymerase Chain Reaction; RSV: Respiratory Syncytial Virus.

^a^ Season as time unit. Rates not comparable among seasons due to the different durations.

^b^ Approximating 1 month = 4.33 weeks.

### 3.2 Description of the seasons

**FIGURE 2:**
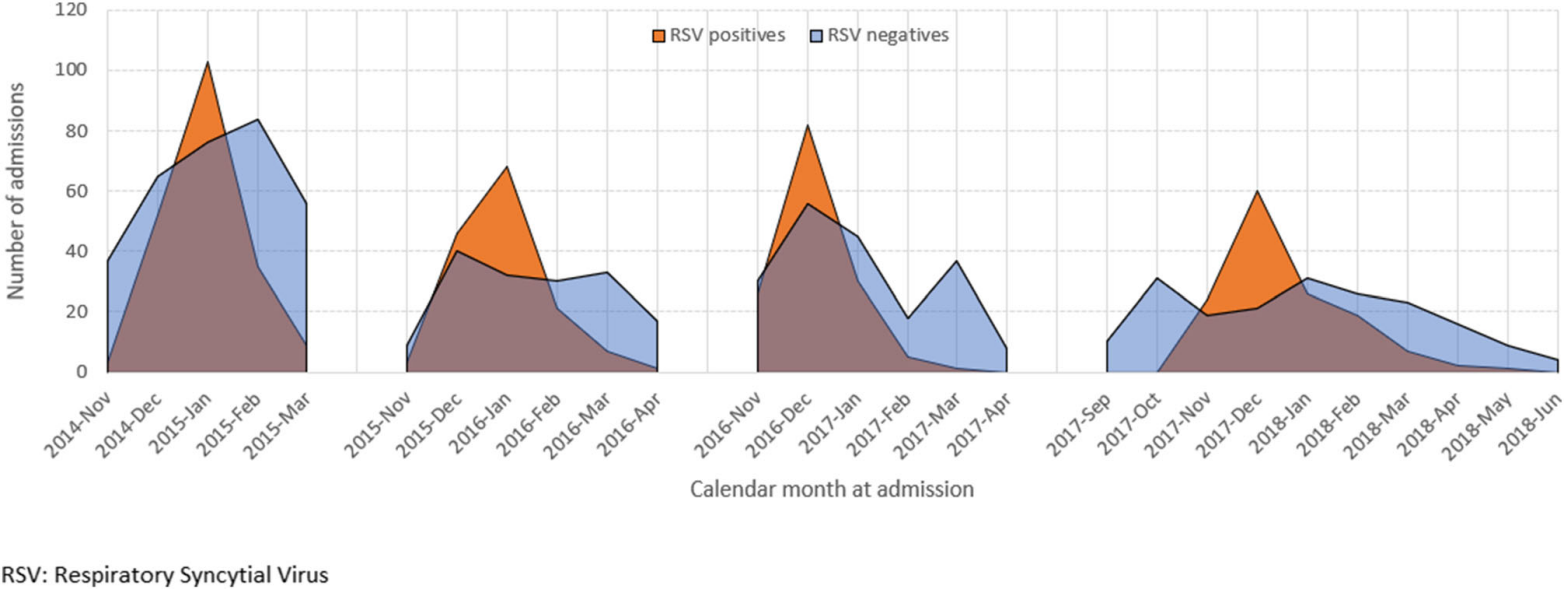
Time distribution of hospitalizations by RSV laboratory result. Infants hospitalized in the VAHNSI network, Valencia Region, Spain

The study was carried out during four consecutive seasons. The RSV circulation period, comprised of weeks 2,014–2,050 to 2,015–2,012, 2,015–2,048 to 2,016–2,016, 2,016–2046 to 2,017–2,010, and 2,017–2,045 to 2,018–2,011 therefore by season, the duration was 16, 20, 17, and 19 weeks, respectively. The peaks were reached in weeks 2,015–2,001, 2,016–2,001, 2,016–2,050 and 2,017–2,052, corresponding to January in 2014/2015 and 2015/2016 and December in 2016/2017 and 2017/2018 (Figure 2).

### 3.3 RSV‐associated hospitalization incidence rates by season, age, and birth month

The overall RSV‐associated hospitalization incidence rates ranged between 1,096.99 (2014/2015) and 1,592.94 (2017/2018) per 100,000 infants‐season (Table 1). When calculating rates per month (to allow comparisons between seasons) during the RSV circulating period, RSV hospitalization incidence rates ranged between 292.46 (2014/2015) to 398.20 (2016/2017) per 100,000 infants‐month (Table 1).

Substantial variability was detected among hospitals, irrespective of the seasons, and among seasons, irrespective of the hospital. For instance, in 2014/2015, rates varied between 83.06 (San Juan Hospital) and 580.93 (General de Castellón Hospital) per 100,000 infants‐month. By season, rates for Doctor Peset Hospital were 116.61, 322.68, 400.63, and 256.97 per 100,000 infants‐month, respectively (Table 1).

**FIGURE 3:**
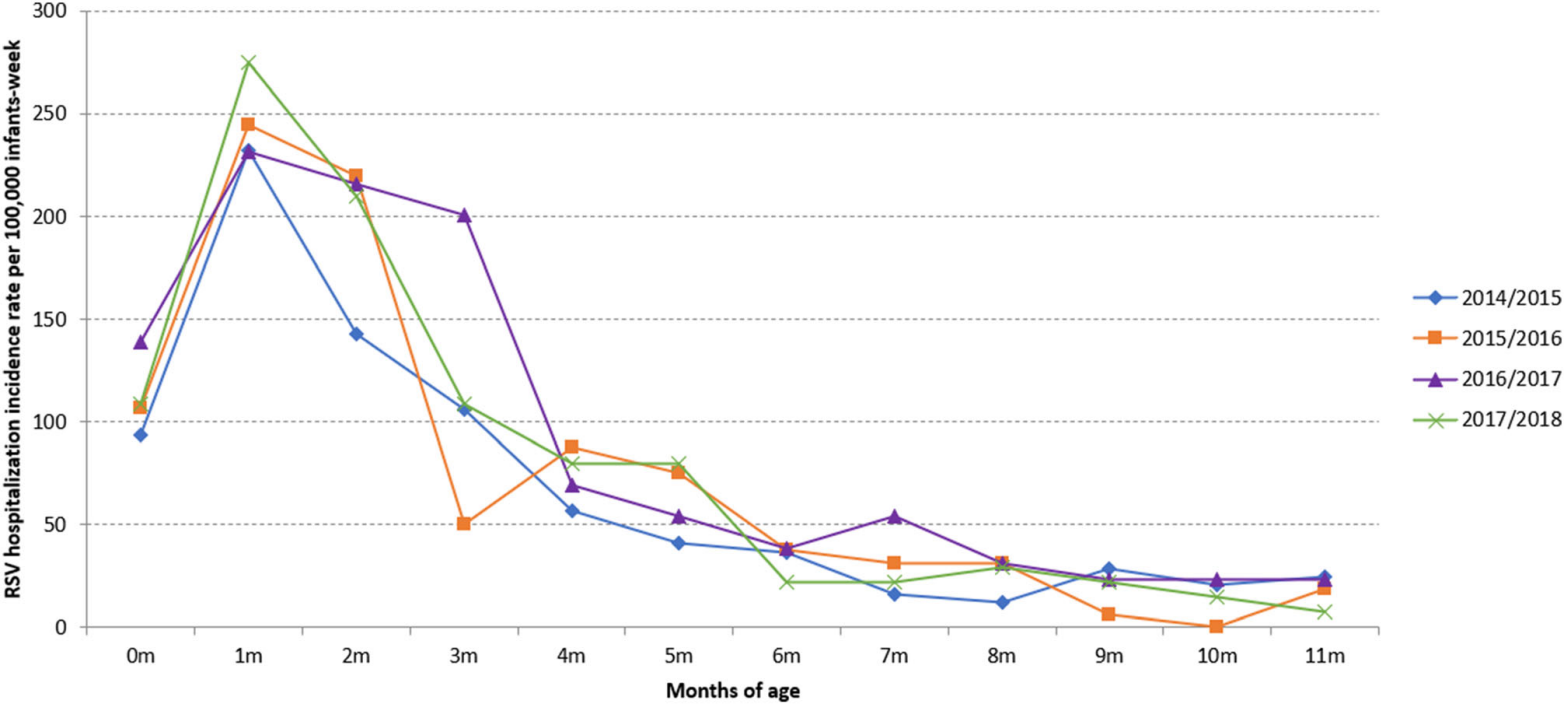
RSV hospitalization incidence rates per 100,000 infants‐week (of RSV circulation) by season and months of age. Infants hospitalized in the VAHNSI network, Valencia Region, Spain

Every season, highest RSV‐associated hospitalization incidence rates were detected in 1 month old infants (ranging between 231.59 and 275.10 per 100,000 infants‐week, in 2016/2017 and 2017/2018, respectively), followed by 2 months old infants (between 142.60 and 216.15 per 100,000 infants‐week, in 2014/2015 and 2016/2017, respectively). RSV‐associated hospitalization incidence rates started to decrease for infants above 2 months old, although it was still high for 3 months old infants (200.71 per 100,000 infants‐week) in 2016/2017 (Figure 3).

**FIGURE 4:**
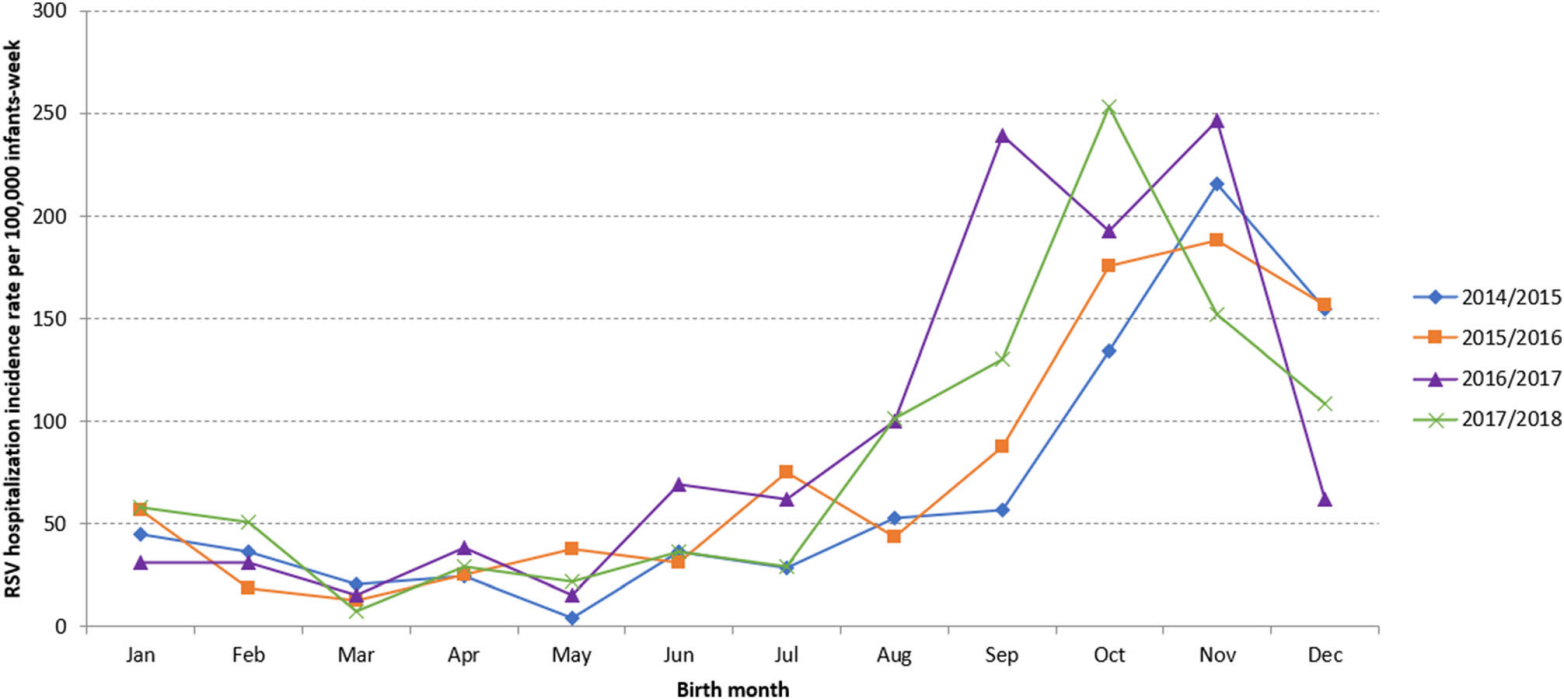
RSV hospitalization incidence rates per 100 000 infants‐week (of RSV circulation) by season and birth month. Infants hospitalized in the VAHNSI network, Valencia Region, Spain

In terms of birth month, highest incidences were found in infants born from August to December, especially from September to November, irrespective of the season (Figure 4). In 2014/2015, 2015/2016, and 2016/2017, highest RSV hospitalization incidence rates were detected in infants born in November (215.94, 188.44, and 247.03 per 100,000 infants‐week, respectively). In 2017/2018, highest rate was in infants born in October (253.38 per 100,000 infants‐week) (Figure 4). Over the four seasons, higher risk of RSV hospitalization was detected in infants born before or at the beginning of the RSV season, and we observed that 54% of infants hospitalized due to RSV in their first RSV season were born outside of the season (April to October) (Table 2).

**TABLE 2:** Characteristics of patients by season and RSV status

	2014/2015					2015/2016					2016/2017					2017/2018				
	RSV+	%	RSV−	%	*p*	RSV+	%	RSV−	%	*p*	RSV+	%	RSV−	%	*p*	RSV+	%	RSV−	%	*p*
Month of birth	202	100	318	100	**0.001**	146	100	161	100	**0.041**	144	100	194	100	**0.017**	139	100	190	100	**<0.001**
September	14	6.93	29	9.12		14	9.59	12	7.45		31	21.53	24	12.37		18	12.95	28	14.74	
October	34	16.83	40	12.58		28	19.18	13	8.07		25	17.36	30	15.46		35	25.18	18	9.47	
November	53	26.24	51	16.04		30	20.55	30	18.63		32	22.22	27	13.92		22	15.83	14	7.37	
December	39	19.31	42	13.21		25	17.12	22	13.66		8	5.56	30	15.46		15	10.79	30	15.79	
January	11	5.45	48	15.09		9	6.16	11	6.83		4	2.78	13	6.70		10	7.19	20	10.53	
February	9	4.46	21	6.60		3	2.05	13	8.07		4	2.78	13	6.70		7	5.04	11	5.79	
March	5	2.48	8	2.52		2	1.37	11	6.83		2	1.39	4	2.06		1	0.72	13	6.84	
April	6	2.97	11	3.46		4	2.74	5	3.11		5	3.47	10	5.15		5	3.60	8	4.21	
May	1	0.50	17	5.35		6	4.11	7	4.35		2	1.39	7	3.61		3	2.16	9	4.74	
June	9	4.46	21	6.60		6	4.11	9	5.59		10	6.94	11	5.67		5	3.60	4	2.11	
July	8	3.96	11	3.46		12	8.22	18	11.18		8	5.56	12	6.19		4	2.88	21	11.05	
August	13	6.44	19	5.97		7	4.79	10	6.21		13	9.03	13	6.70		14	10.07	14	7.37	
Age at admission					0.760					**0.002**					0.521					**0.006**
0 months	23	11.39	37	11.64		17	11.64	18	11.18		18	12.50	19	9.79		15	10.79	19	10.00	
1 month	58	28.71	95	29.87		39	26.71	35	21.74		30	20.83	50	25.77		39	28.06	40	21.05	
2 months	35	17.33	52	16.35		35	23.97	21	13.04		28	19.44	30	15.46		31	22.30	25	13.16	
3 months	26	12.87	28	8.81		8	5.48	18	11.18		26	18.06	21	10.82		15	10.79	14	7.37	
4 months	15	7.43	17	5.35		14	9.59	9	5.59		10	6.94	16	8.25		11	7.91	15	7.89	
5 months	10	4.95	17	5.35		13	8.90	12	7.45		7	4.86	13	6.70		11	7.91	9	4.74	
6 months	9	4.46	16	5.03		6	4.11	9	5.59		5	3.47	13	6.70		3	2.16	12	6.32	
7 months	4	1.98	18	5.66		5	3.42	9	5.59		7	4.86	6	3.09		3	2.16	13	6.84	
8 months	3	1.49	6	1.89		5	3.42	4	2.48		4	2.78	7	3.61		4	2.88	10	5.26	
9 months	7	3.47	14	4.40		1	0.68	15	9.32		3	2.08	4	2.06		3	2.16	10	5.26	
10 months	5	2.48	8	2.52		0	0.00	7	4.35		3	2.08	8	4.12		2	1.44	8	4.21	
11 months	7	3.47	10	3.14		3	2.05	4	2.48		3	2.08	7	3.61		2	1.44	15	7.89	
Prematurity					0.058					0.141					0.403					0.781
<29 weeks	0	0.00	6	1.89		0	0.00	1	0.62		0	0.00	2	1.03		0	0.00	1	0.53	
29 to <37 weeks	22	10.89	24	7.55		26	17.81	18	11.18		12	8.33	22	11.34		16	11.51	25	13.16	
≥37 weeks	179	88.61	286	89.94		119	81.51	140	86.96		132	91.67	170	87.63		123	88.49	160	84.21	
Breastfeeding					0.951					0.704					0.081					0.360
No	63	31.19	100	31.45		46	31.51	54	33.54		51	35.42	87	44.85		48	34.53	75	39.47	
Yes	139	68.81	218	68.55		100	68.49	107	66.46		93	64.58	107	55.15		91	65.47	115	60.53	
Comorbidities																				
None	187	92.57	299	94.02	0.514	141	96.58	157	97.52	0.741	120	96.57	174	89.69	0.086	137	98.56	178	93.68	**0.030**
Chronic cardiovascular disease	1	0.50	5	1.57	0.412	2	1.37	2	1.24	>0.999	3	2.08	7	3.61	0.526	0	0.00	7	3.68	**0.023**
COPD, bronchitis or another ≠ asthma	10	4.95	7	2.20	0.086	1	0.68	0	0.00	0.476	17	11.81	9	4.64	**0.014**	0	0.00	0	0.00	NA
Anemia	0	0.00	1	0.31	>0.999	0	0.00	0	0.00	NA	3	2.08	1	0.52	0.316	0	0.00	1	0.53	>0.999
Renal impairment	3	1.49	4	1.26	>0.999	0	0.00	1	0.62	>0.999	0	0.00	2	1.03	0.509	1	0.72	0	0.00	0.422
Contact with kids					0.117					0.558					0.123					0.702
No	83	41.09	109	34.28		40	27.40	49	30.43		49	34.03	51	26.29		39	28.06	57	30.00	
Yes	119	58.91	209	65.72		106	72.60	112	69.57		95	65.97	143	73.71		100	71.94	133	70.00	
Kindergarten/school					0.213					0.123					0.594					0.076
No	188	93.07	304	95.60		141	96.58	149	92.55		139	96.53	185	95.36		133	95.68	172	90.53	
Yes	14	6.93	14	4.40		5	3.42	12	7.45		5	3.47	9	4.64		6	4.32	18	9.47	
Smokers at home					0.215					0.207					**0.003**					0.124
No	118	58.42	203	63.84		85	58.22	105	65.22		110	76.39	119	61.34		85	61.15	100	52.63	
Yes	84	41.58	115	36.16		61	41.78	56	34.78		34	23.61	75	38.66		54	38.85	90	47.37	

*Note*: Infants hospitalized in the VAHNSI network, Valencia Region, Spain.

Abbreviations: COPD, Chronic Obstructive Pulmonary Disease; NA, not applicable; RSV, Respiratory Syncytial Virus.

### 3.4 Risk factor of infants hospitalized with laboratory confirmed RSV

A significant difference was observed based on birth month between RSV positive and RSV negative cases in all seasons (*p* values = 0.001, 0.041, 0.017, and <0.001, by season). Age at admission was also substantially different between RSV positives and negatives in 2015/2016 and 2017/2018 (*p* values = 0.002 and 0.006, respectively). The RSV positivity percentage (12–14%) was half of the RSV negative percentage (30–36%) in infants 6 months of age or above in 2015/2016 and one third in 2017/2018. Neither prematurity nor breastfeeding showed an association with RSV positive cases compared to RSV negative cases in any season. Contact with other kids or attendance to kindergarten did not show an association with RSV positivity. The presence of smokers at home was associated with RSV negativity in the 2016/2017 season (*p* value = 0.003). Twenty‐four percent of RSV positive cases were exposed to tobacco versus 39% of RSV negative cases (Table 2).

### 3.5 Impact of age on RSV‐associated hospitalization

**FIGURE 5:**
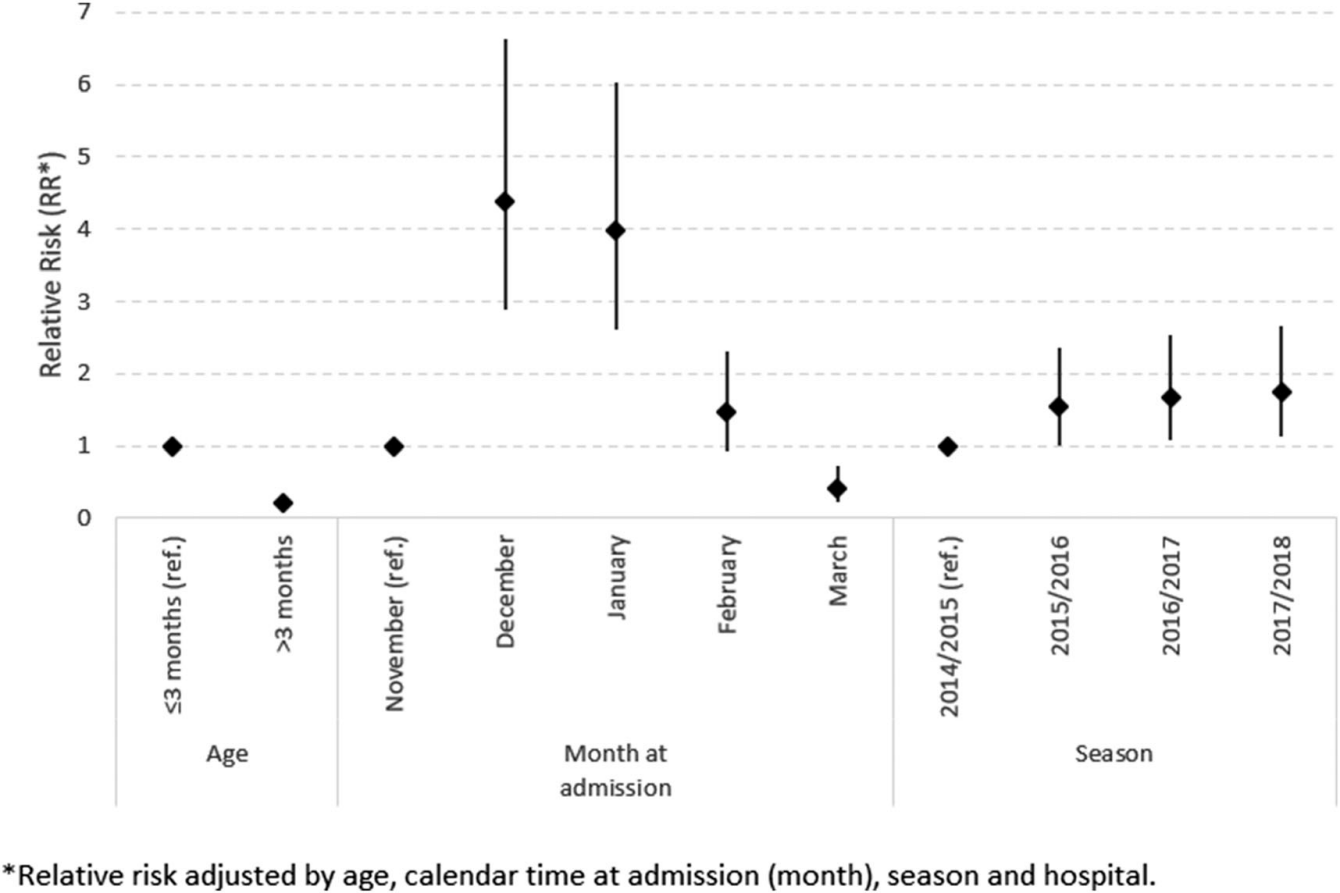
Adjusted relative risk (RR) of RSV infection. Infants hospitalized in the VAHNSI network, Valencia Region, Spain

The results of the Negative Binomial regression revealed that the adjusted relative risk (aRR) of RSV decreased by 78% (aRR = 0.22, 95% CI [0.17–0.29]) in infants >3 months of age in comparison to infants ≤3 months of age. There was a higher RSV‐associated hospitalization risk in December and January (December: aRR = 4.37, 95% CI [2.88–6.63] and January: aRR = 3.97, 95% CI [2.61–6.04]) as compared to November. The risk decreased significantly in March (aRR = 0.41, 95% CI [0.23–0.73]), with 59% of risk reduction as compared to November. By season, the highest risk RSV‐associated hospitalization was found in the 2017/2018 (aRR = 1.75, 95% CI [1.14–2.67]), followed by 2016/2017 (aRR = 1.66, 95% CI [1.08–2.53]) and 2015/2016 (aRR = 1.55, 95% CI [1.01–2.37]), considering 2014/2015 as reference (Figure 5).

### 3.6 RSV‐associated disease—RSV positivity rate according to ICD‐10 discharge diagnoses by season and age

Over the four seasons, 601 infants with an ICD‐10 LRI code (J09‐J22) were RSV laboratory confirmed, which represents 51% of the infants included in the study with this discharge code. This positivity rate varied from 48% to 57%, depending on the season. From the infants with an ICD‐10 discharged code related to bronchiolitis (J21, *N* = 872), 58% (*N* = 505) were RSV laboratory confirmed: from 53% (*N* = 127) to 66% (*N* = 111), by season. From the total infants discharged with an ICD‐10 code related to pneumonia (J12‐J18, *N* = 100), 39% (*N* = 39) were laboratory confirmed for RSV: from 18% (*N* = 3) to 60% (*N* = 18), by season. RSV positivity rates were provided by discharged ICD‐10 code and age in Table 3.

**TABLE 3:** RSV positivity rate by ICD code at discharge, months of age, and season

Surveillance period	Months	Lower respiratory infection (J09‐J22)			Bronchiolitis (J21)			Pneumonia (J12‐J18)		
		*N*	RSV+	%	*N*	RSV+	%	*N*	RSV+	%
2014/2015 (21 weeks, 4.8 months)	[0–3]	221	108	48.87	178	97	54.49	10	4	40.00
	[3–6]	92	49	53.26	73	42	57.53	5	1	20.00
	[6–12]	83	33	39.76	40	17	42.50	13	5	38.46
	All	396	190	47.98	291	156	53.61	28	10	35.71
2015/2016 (24 weeks, 5.5 months)	[0–3]	148	89	60.14	109	73	66.97	5	2	40.00
	[3–6]	59	33	55.93	43	27	62.79	6	2	33.33
	[6–12]	54	19	35.19	23	11	47.83	14	4	28.57
	All	261	141	54.02	175	111	63.43	25	8	32.00
2016/2017 (23 weeks, 5.3 months)	[0–3]	153	76	49.67	134	69	51.49	1	0	0.00
	[3–6]	88	43	48.86	74	41	55.41	5	1	20.00
	[6–12]	58	25	43.10	30	17	56.67	11	2	18.18
	All	299	144	48.16	238	127	53.36	17	3	17.65
2017/2018 (41 weeks, 9.5 months)	[0–3]	115	79	68.70	99	72	72.73	9	8	88.89
	[3–6]	58	35	60.34	43	30	69.77	6	6	100.00
	[6–12]	48	12	25.00	26	9	34.62	15	4	26.67
	All	221	126	57.01	168	111	66.07	30	18	60.00
2014/2018	[0–3]	637	352	55.26	520	311	59.81	25	14	56.00
	[3–6]	297	160	53.87	233	140	60.09	22	10	45.45
	[6–12]	243	89	36.63	119	54	45.38	53	15	28.30
	All	1,177	601	51.06	872	505	57.91	100	39	39.00

*Note*: Children <1 years old hospitalized in the VAHNSI network, Valencia Region, Spain.

Abbreviation: RSV, Respiratory Syncytial Virus.

## 4 DISCUSSION

In this study, we explored RSV laboratory‐confirmed hospitalizations in infants <1 year old in a prospective, active‐surveillance, multicenter network in Valencia Region (Spain) during four consecutive seasons from 2014/2015 to 2017/2018.

Our data revealed a general common seasonality of RSV, between November and March, with high circulation in December–January every year. The seasonal pattern observed in our study is the same as described by others in temperate regions.^6^, ^20^, ^21^ This pattern was observed even in 2017/2018 when the surveillance was extended from September to June. However, after in depth analysis, we observed that the RSV season moved forward every year, but with slight difference on the start of the season for the last two seasons (2016/2017 and 2017/2018) and an earlier peak of RSV in comparison with the two previous years. This result is of importance for the development of future immunization strategies targeting infants with a defined efficacy period^22^ and, therefore, should be continuously monitored to identify the best timing to implement these strategies.

Overall, the RSV A subtype was most detected, although subtype B prevailed in 2016/2017. Similar studies have also detected the RSV A subtype predominance over time.^11^

As the RSV circulation period did not last the same for all seasons (from 16 to 20 weeks), we calculated the RSV hospitalization incidence rates by week, obtaining the lowest rate in the 2014/2015 season and the highest in 2016/2017. After adjusting by age, calendar time, season and hospital, the lowest risk was still detected in 2014/2015, and the highest risk was in 2017/2018. Our study demonstrates RSV‐associated hospitalization incidence variability from season to season. Similar observation was made in other studies,^6^ demonstrating the unpredictability of the severity of RSV‐associated disease.

In the effort to better characterize infants hospitalized due to RSV, we found that majority of infants are full terms and otherwise healthy, which is in agreement with previous publications.^23^, ^24^ In addition, we observed that the month of birth and age were critical factors for hospitalization due to RSV. Highest rates were detected in young ones and in those born before or at the beginning of the RSV season. Previous studies also reported the same findings.^6^, ^20^, ^25^, ^26^, ^27^, ^28^, ^29^ RSV‐associated hospitalization risk in infants born at the end of RSV season (January to March) appeared to be lower than the ones born between August and September. Transfer of maternal antibodies certainly has a role here.^30^ However, this protection appears to be short with the RSV‐associated hospitalization incidence peak in infants at 1 month old, regardless of the season. The incidence rates started to decrease for infants >2 months old, although it remained quite high in infants with 3 months of age in 2016/2017.

Another important finding of this study is the percentage of laboratory‐confirmed RSV per ICD‐10 code. RSV has been reported as the main cause of bronchiolitis in infants in several publications.^28^, ^31^, ^32^ We detected RSV in 58% of the infants discharged from hospital with a bronchiolitis ICD‐10 code, in 51% of LRI and in 39% of pneumonia, with variations based on season and age. Hospital data registries are commonly used to estimate RSV‐associated hospitalizations at national or regional level.^5^, ^9^, ^33^ However, hospitalized patients are tested for RSV at clinician's discretion. Therefore, some RSV cases could be missed, leading to an RSV misclassification.^9^ By contrast, the VAHNSI network tested by RT‐PCR any admitted patient with a suspicion of a respiratory infection. Then, our laboratory‐confirmed information could support better ascertainment of RSV‐associated hospitalization by adjusting results from ICD‐10 code to the positivity rates described in our data. Another method of adjustment was performed by Arriola et al.^23^ in United‐States that account for testing practices and test method sensitivities.

The role of other respiratory viruses on RSV infections has been previously analyzed.^11^, ^34^ We detected the presence of co‐infections due to other respiratory viruses among 13% of RSV‐positive cases. Other studies reported similar percentages, ranging between 10% and 16%.^23^, ^35^ In our study, the commonest mixed infection was the combination of RSV and rhinovirus/enterovirus. This is in agreement with other publications,^11^, ^36^ although different combinations were more frequent in other studies, as Bonzel et al.^35^ reported, RSV and human bocavirus as the most common detected coinfection. No agreement has been reached regarding the severity of the RSV mixed infections. Whereas some studies reported a more severe course in patients with RSV coinfection,^37^ others did not find differences in the clinical severity between RSV single infections and coinfections.^38^

This work presented some limitations. The different durations of the seasons and the variability among participating hospitals policies can make comparisons hard to interpret. We approached the denominators for each month of age by dividing the total population under 1 year old by 12 months. Although it was a satisfactory approach, we lost some precision in the estimates.

Despite these limitations, our study was robust by using laboratory confirmed data and a well‐defined catchment population. We provided recent data from four consecutive seasons from a hospital network with large experience in observational studies, using laboratory‐confirmed RSV cases and the same testing practice in a centralized laboratory. Very few networks presented these methodologies^24^, ^39^ and, in some studies, different specimens and diagnostic assays were used at site level, mixing commercial and institution‐specific in‐house RT‐PCR in the same network.^24^

Our catchment population was very well defined, so we had the possibility of calculating hospitalization incidence rates to estimate the burden of the disease, providing more precise estimates than those obtained when using non‐population‐based study.^39^, ^40^ Finally, we were able to link clinical, demographic and laboratory data to improve the characterization of RSV‐associated hospitalizations in infants.

## 5 CONCLUSIONS

There is a lack of prospective, hospital‐based, active‐surveillance epidemiological studies on RSV thereby limiting the knowledge on how this pathogen impacts infant health. The VAHNSI network is a crucial component required to inform and to support prospective immunization that will occur in near future to protect infants at risk of RSV. This study brings forth the unpredictability of RSV epidemics, the critical impact of age, and the comparable distribution of RSV‐associated hospitalization among infants born outside or during the RSV season. There is critical need for solutions to decrease the burden of RSV on health resources consumption which is driven by otherwise healthy infants. This study provides some views to support decision makers to decrease hospitals overcrowding associated with RSV infection.

## AUTHOR CONTRIBUTIONS

**Ainara Mira‐Iglesias:** Conceptualization; data curation; formal analysis; funding acquisition; methodology; project administration; validation. **Clarisse Demont:** Conceptualization; methodology. **F. Xavier Lopez‐Labrador:** Conceptualization; formal analysis; methodology. **Beatriz Mengual‐Chuliá:** Formal analysis. **Javier García‐Rubio:** Data curation; project administration. **Mario Carballido‐Fernandez:** Investigation. **Miguel Tortajada‐Girbes :** Investigation. **Juan Mollar‐Maseres:** Investigation. **Germán Schwarz‐Chavarri:** Investigation. **Joan Puig‐Barbera:** Conceptualization; validation. **Javier Díez‐Domingo:** Conceptualization; funding acquisition; methodology; supervision; validation.

## FUNDING INFORMATION

This collaborative study has been partly funded by FISABIO‐Public Health (Fundación para el Fomento de la Investigación Sanitaria y Biomédica de la Comunitat Valenciana), Sanofi Pasteur, and CIBER‐ESP (ISCIII).

## PATIENT CONSENT STATEMENT

All patients signed a written informed consent before their inclusion in the study.

## Supporting information


**Table S1:** Admission diagnoses for children <1 year old in the Valencia Hospital Network for the Study of Influenza (VAHNSI).

## Data Availability

The data that support the findings of this study are available from the corresponding author upon reasonable request.
