# Supplementary material for: Role of age and birth month in infants hospitalized with RSV‐confirmed disease in the Valencia Region, Spain
Source: Influenza Other Respir Viruses. 2021 Nov 24;16(2):328–39. doi: 10.1111/irv.12937 (PMC8818825; doi:10.1111/irv.12937)
Supplement: Supplementary file 1 — Table S1: Admission diagnoses for children <1 year old in the Valencia Hospital Network for the Study of Influenza (VAHNSI). [file IRV-16-328-s001.docx]

| **Eligibility diagnoses, symptoms and signs** | **ICD 10 Codes** |
| --- | --- |
| Acute respiratory infection | J00-J06, J20-J22, H66.90 |
| Asthma | J45.2-J45.22, J45.9-J45.998, J44-J44.9 |
| Heart failure | I50-I50.9, I51.4 |
| Pneumonia and influenza | J09-J18 |
| Myalgia | M79.1 |
| Altered consciousness, convulsions, febrile convulsions | R40.0, R40.1, R40.20, R40.4, R56.00, R56.01 |
| Dyspnoea / respiratory abnormality / shortness of breath / respiratory symptoms | R06-R06.9 |
| Fever or fever of unknown origin or non-specified | R50, R50.9 |
| Cough | R05 |
| Apnoea | R06.81 |
| Sepsis, Systemic inflammatory response syndrome | A41.9, R65.10, R65.11, R65.20 |

**Supplementary Table 1**: Admission diagnoses for children <1 year old in the Valencia Hospital Network for the Study of Influenza (VAHNSI).
